# Daily dose of metformin caused acute kidney injury with lactic acidosis: a case report

**DOI:** 10.1186/s13256-023-04136-0

**Published:** 2023-09-16

**Authors:** Maho Ariga, Junichiro Hagita, Midori Soda, Yasuhisa Oida, Hitomi Teramachi, Kiyoyuki Kitaichi

**Affiliations:** 1 Laboratory of Pharmaceutics, Department of Biomedical Pharmaceutics, Gifu Pharmaceutical University, 1-25-4 Daigakunishi, Gifu, 501-1196 Japan; 2 Kariya Toyota General Hospital, Aichi, Japan; 3 Laboratory of Clinical Pharmacy, Department of Pharmacy Practice and Science, Gifu Pharmaceutical University, Gifu, Japan

**Keywords:** Metformin, Acute kidney injury, Diabetes, Case report, Lactic acidosis

## Abstract

**Background:**

Metformin-induced lactic acidosis with acute kidney injury is rare but well known. Here we report a case of a Japanese patient taking metformin who experienced severe acute renal failure accompanied with significantly elevated metformin plasma concentrations and signs of lactic acidosis.

**Case presentation:**

A 60-year-old Japanese man with type II diabetes, who was taking metformin (500 mg three times a day) along with several other medications, visited the emergency department with dizziness, malaise, and oliguria. The initial laboratory test results showed elevated levels of serum creatinine and blood urea nitrogen, although his renal function was normal approximately 2 weeks earlier. His lactate level was raised (4.27 mmol/L), and he was diagnosed with lactic acidosis. Considering the low creatinine clearance and elevated urinary albumin/serum creatinine ratio, urinary *N*-acetyl-β-d-glucosaminidase level, and β2-microglobulin level, the patient was further diagnosed with AKI (in other words, acute tubular necrosis). A renal biopsy performed on day 3 after admission revealed renal tubular epithelium necrosis, supporting this diagnosis. The patient underwent intermittent hemodialysis until he was discharged on day 13. The metformin concentrations on days 3, 5, and 7 were 8.95, 2.58, and 0.16 μg/mL, respectively, which is significantly higher than the maximal steady-state concentration of metformin at the recommended dosage (approximately 1 μg/mL). The calculated pharmacokinetic parameters of metformin suggested poor renal excretion and a low distribution volume at higher metformin levels. Other possible acute kidney injury-causing factors included dehydration, alcohol consumption, and the use of an angiotensin receptor blocker or SGLT2 inhibitor.

**Conclusions:**

This is the first reported case of acute kidney injury possibly caused by high levels of metformin with lactic acidosis in a patient treated with the recommended metformin dose. Thus, the development of metformin-induced acute kidney injury should be considered for patients with several acute kidney injury risk factors who are taking metformin.

## Background

Metformin (Met) is a biguanide used to treat type II diabetes mellitus (T2DM). It inhibits gluconeogenesis in the liver, decreases glucose absorption from the intestine, and promotes the utilization of glucose in the peripheral tissues by activating adenosine monophosphate (AMP)-activated protein kinase [1]. Met is frequently used to treat T2DM as it is relatively safer than other drugs. However, one of the major adverse effects of Met is lactic acidosis with an occurrence rate that is lower than 10 in 100,000 patients per year [2]. Here, we report a case of a Japanese patient taking Met who experienced severe acute renal failure and acute kidney injury (AKI) accompanied with significantly elevated Met plasma concentrations and lactic acidosis.

## Case presentation

A 60-year-old Japanese man with a 9-year history of T2DM presented at the emergency department of Kariya Toyota General Hospital with anuria and severe general fatigue. On admission, the patient was transported by ambulance due to his difficulty in moving. On evaluation, the patient reported fatigue, malaise, weight loss, and intermittent nausea and vomiting. He reported no headaches, joint, or muscle pain. He denied having a fever or contact with another sick individual.

Apart from T2DM, his past medical history included hypertension and lumbar disc herniation. He had no history of psychiatric disease and no known drug allergies. He had no family history of diabetes mellitus, lived alone, and his educational status was not obtained. The patient had smoked 1 pack of cigarettes per day for 45 years and consumed approximately 540 mL of Japanese sake daily. His medications included Met (500 mg three times daily), sitagliptin (50 mg once daily), pioglitazone (15 mg once daily), dapagliflozin (5 mg once daily), amlodipine (5 mg once daily), azilsartan/amlodipine (20 mg/5 mg once daily), rosuvastatin (2.5 mg once daily), and a hydrogel patch containing loxoprofen sodium.

Renal function was normal before admission and the Met dosage used to treat T2DM was suitable for this patient. No abnormality was found in the patient’s blood tests (data not shown) when he had visited his doctor 13 days before admission. Notably, 12 days before admission, his work had changed from office to physical work (approximately 30,000–40,000 steps per day). He had noted a gradual decrease in urinary output for several days before admission, but had no other symptoms. Two days before admission, he developed anuria, dizziness, and nausea.

**Fig. 1:**
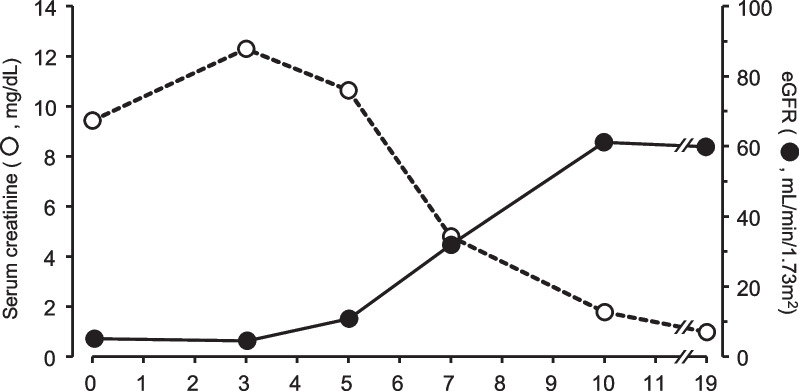
Time-course changes in serum creatinine and estimated glomerular filtration rate (eGFR)

The initial laboratory findings are shown in Table 1. The time-course changes in serum creatinine and blood urea nitrogen (BUN) levels after admission are shown in Fig. 1. The lactate concentration of the patient was 4.27 mmol/L and the arterial pH was 7.31. Considering the previously established definition for lactic acidosis (lactate > 5 mmol/L and pH < 7.35) [3], the patient did not meet these criteria. However, a recently updated definition of lactic acidosis revealed that this patient taking Met fulfilled the criteria for the diagnosis of lactic acidosis because the lactate concentration was > 4 mmol/L [4].

**Table 1:** Laboratory findings of patient on admission

Variable	Values	References
Hematological parameters		
White blood cells (per μL)	8600	3300–8600
Hemoglobin (g/dL)	13.5	11.6–14.8
Platelets (/μL)	214,000	158,000–348,000
Biochemical parameters		
Sodium (mmol/L)	135	135–140
Potassium (mmol/L)	4.4	4.1–5.1
Cloride (mmol/L)	92	101–108
Blood urea nitrogen (mg/dL)	71	8.0–20
Creatinine (mg/dL)	9.44	0.46–0.79
Estimated glomerular filtration rate (mL/min/1.73 m^2^)	5.1	
Calcium (mg/dL)	8.7	8.5–10.0
Phosphorus (mg/dL)	11.0	2.5–4.5
Uric acid (mg/dL)	10.2	3.5–7.2
Glucose (mg/dL)	129	70–110
Total bilirubin (mg/dl)	0.4	0.4–1.5
Aspartate aminotransferase (U/L)	23	8–25
Alanine aminotransferase (U/L)	17	9–32
Alkaline phosphatase (U/L)	174	7–33
Creatine kinase (U/L)	715	59–248
Lactate dehydrogenase (U/L)	233	124–222
Total protein (g/dL)	6.9	6.6–8.1
Albumin (g/dL)	4.1	4.1–5.1
C-reactive protein (mg/dL)	0.56	0.0–0.14
Hemoglobin A1c (%)	6.3	< 5.8
Immunologic parameters		
MPO-ANCA (U/mL)	< 3.5	< 3.5
PR3-ANCA (U/mL)	< 3.5	< 3.5
Anti GBM antibody(U/mL)	< 7.0	< 7.0
ANA (titer)	< 40	< 40
Arterial blood gases		
pH	7.316	7.35–7.45
Partial pressure of carbon dioxide (mmHg)	28.3	35.0–45.0
Partial pressure of oxygen (mmHg)	89.9	> 80
Bicarbonate (mmol/L)	14.1	22–28
Anion gap	23.5	10–14
Lactic acid (mmol/L)	4.27	< 2
Urinalysis test		
pH	6.0	4.5–8.0
Protein	3+	–
Occult blood	3+	–
Glucose	–	–
Ketone	2+	–
Urine red blood cell (per HPF)	50–99	< 1–4
Urine white blood cell (per HPF)	1–4	< 1–4
Hyaline cast (/HPF)	5–9	< 5–9
Urinary protein (g/g Cre)	11.04	< 0.15
NAG (U/gCre)	420.3	1.6–6.3
Urinary β2-MG (μg/g Cre)	149	< 200

ANA; anti-nuclear antibody, GBM; glomerular basement membrane, MG; microglobulin, MPO-ANCA; myeloperoxidase-anti-neutrophil cytoplasmic antibodies, NAG; N-acetyl-β-d-glucosaminidase, PR3-ANCA; proteinase 3-ANCA

The physical examination at admission indicated the following: height of 172.3 cm, weight of 69.3 kg, blood pressure of 159/94 mmHg, regular pulse of 96 beats per minute, body temperature of 36.3 °C, and oxygen saturation of 99% breathing ambient air. There was no sign of hypoglycemia since blood glucose was 129 mg/dL (Table 1). This case is considered to be different from either diabetic ketoacidosis or euglycemic diabetic ketoacidosis. Despite the presence of normal glucose levels, he was not in the insulin-dependent state characteristic of euglycemic diabetic ketoacidosis and did not present with the physical symptoms of ketosis (tachypnea, nausea, vomiting, abdominal pain, and impaired consciousness). The patient was alert and appeared well. Cardiac auscultation revealed a regular rhythm without murmurs or gallops and an audible S1 and S2. Respiratory sounds were clear to auscultation with no crackles, wheezes, or bronchial breath sounds. The abdomen was non-distended, soft, and non-tender. His legs had no edema or muscle weakness, and the remainder of the examination was unremarkable.

A radiograph of the chest was normal. Computed tomography (CT) showed slight cortical atrophy in the bilateral kidneys; however, no kidney stone or hydronephrosis was observed. There was irregular thickening of the small bowel wall without adjacent fat stranding and no sign of inferior vena cava collapse.

Bilateral hydronephrosis and nephrolithiasis were excluded based on the CT scan data. Glomerulonephritis was also excluded based on the negative autoantibody findings. The ratio of urea nitrogen to creatinine was < 20, suggesting a possible cause for the intrinsic renal injury. There was no hematuria because the presence of red cells in the urine (Table 1) was due the trauma induced by urinary bladder catheterization on admission. Considering the low estimated glomerular filtration rate (eGFR) and high urinary protein/creatinine ratio (11.04 g/g Cre), urinary *N*-acetyl-β-d-glucosaminidase (420.3 U/g Cre), and β2-microglobulin/creatinine ratio (149 µg/g Cre) (Table 1), the patient was presumptively diagnosed with acute tubular necrosis with metabolic acidosis (anion-gap 23.5 mmol/L). Renal pathology findings on day 3 after admission revealed that 3 of 23 glomeruli were sclerotic and no significant pathological alterations were observed with light microscopy. However, the proximal tubular cells were diffusely enlarged with vacuolar degeneration. The same pathology may be seen with Fabry’s disease and use of osmotic diuretics, but the patient’s history was negative. All immunofluorescence results were negative and, apart from the vacuolization and swelling of the proximal tubules, no obvious abnormal findings were observed by electron microscopy. Based on the pathological findings and clinical course, the diagnosis of acute tubular necrosis was confirmed.

After admission, intravenous hydration therapy was started. On the day of presentation at the emergency unit (day 0), he was hospitalized and hemodialysis (HD) was introduced to remove Met and improve acid–base disruption and anuria. In detail, HD was performed with a polysulfone dialyzer [Pinnafine® PN-100 (filter size 1.0  m^2^, Fresenius Medical Care AG and Co., Hessen, Germany)﻿ (at day 0, 1, and 3 with small molecule heparin) or Pinnafine® PN-140  (filter size 1.4  m^2^, Fresenius Medical Care AG and Co., Hessen, Germany) (at day 5 and 7 with Nafamostat Mesilate)] with blood and dialysate (Kidaly 4E solution, Fuso Pharmaceutical Industries, Ltd., Osaka, Japan) flow rates of 150 and 500 mL/min, respectively, for 3 hours per session. During the HD session, the patient’s condition was stable. The eGFR was calculated from serum creatinine levels on days 0, 3, 5, 7, 10, and 19 were 5.1, 4.5, 10.8, 31.9, 61.2, and 59.9 mL/min/1.73 m^2^, respectively (Fig. 1). The patient resumed urination on day 2. On day 5, anuria was recovered after the third session of HD. Renal function gradually improved, and on day 11, the serum creatinine level reached 1.78 mg/dL. The patient was discharged 13 days after hospitalization, and no major prognostic problems were observed.

**Fig. 2:**
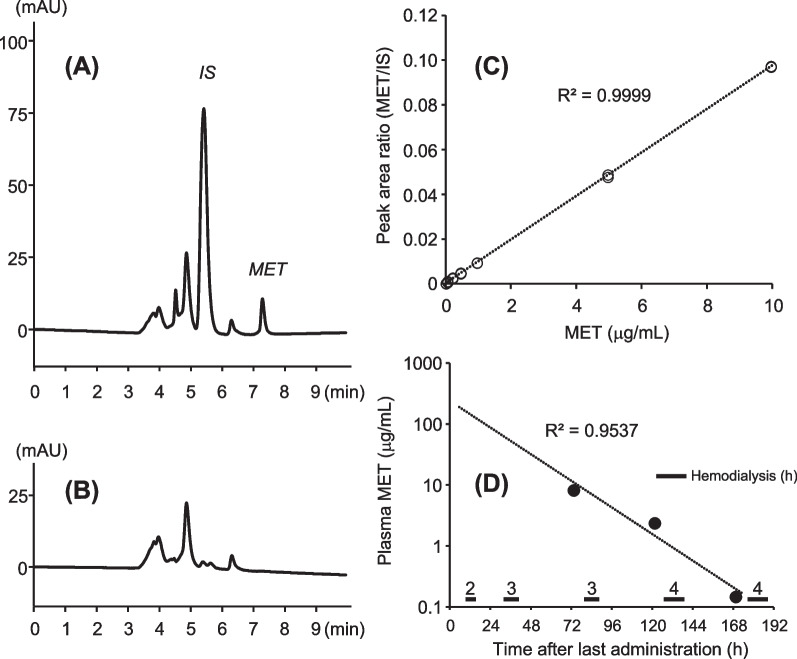
Typical chromatograms, calibration curve, and plot of the observed Met plasma levels in the patient. Typical chromatograms of **A** plasma spiked with Met (10 μg/mL) and internal standard (250 μg/mL) and **B** blank plasma. **C** Standard calibration curve of Met (*N* = 2). **D** Plot of the observed Met plasma levels in the patient

The plasma concentrations of Met were measured using high-performance liquid chromatography (HPLC)–ultraviolet (UV) as per a previous report [5], with slight modification and in accordance with the validation criteria guidelines of the US Food and Drug Administration [6]. The Met concentrations on days 3, 5, and 7 were 8.95, 2.58, and 0.16 µg/mL, respectively. The semilogarithmic concentration–time plots for Met showed good linearity (*R*^2^ = 0.954, Fig. 2D). The one-compartment model pharmacokinetic (PK) analysis yielded an observed *k*_e (day 3–7)_ of 0.04 hours^−1^ and calculated *t*_1/2 (day 3–7)_ of 16.5 hours. Moreover, the *t*_1/2 (day 3–5)_ was 26.7 hours, and *t*_1/2 (day 5–7)_ was 11.9 hours.

## Discussion and conclusions

This is a case where a patient was being treated with the recommended Met dose and presented at the emergency unit and was diagnosed with AKI. PK analysis revealed that AKI might be caused by high plasma Met levels, consistent with previous reports that have demonstrated that Met overdose causes AKI [7–9]. Possible mechanism to cause AKI by Met is the decrement of mitochondrial adenosine triphosphate (ATP) by Met [10] since AKI can arise from mitochondrial ATP depletion [11]. Although it is well known that a daily dose of Met exerts renal protecting effects [10, 12–14], these results suggest that a daily dose of Met could cause AKI, particularly in patients with several AKI risk factors.

The patient had severe renal failure with oliguria on the day of hospitalization (day 0), although laboratory data obtained 13 days earlier indicated normal renal function. Based on three data points of Met plasma concentration up to 7 days after hospitalization, the *t*_1/2_ of Met was 16.5 hours. This *t*_1/2_ is comparable to that of patients with severe renal failure (creatine clearance rate: 10–30 mL/minute) [15]. However, in this patient, the *t*_1/2 (day 3–5)_ and *t*_1/2 (day 5–7)_ were 26.7 and 11.9 hours, respectively. Considering that the patient underwent 5 hours of HD between days 0 and 3, 3 hours between days 3 and 5, and 4 hours between days 5 and 7, the actual *t*_1/2_ would have been far worse. However, slight renal function recovery occurred after day 5, which is consistent with the eGFR recovery.

Regarding the value of *t*_1/2 (day 3–5)_, the one-compartment analysis yielded an estimated plasma Met concentration on the day of hospitalization (*C*_0_) of 58 µg/mL. This value is notably higher since healthy volunteers receiving the recommended Met daily dose (500 mg three times a day) had a Met *C*_max_ at a steady-state (*C*_ss,max_) of approximately 1.0 µg/mL [16]. This high Met concentration occurred in a patient who attempted suicide by taking 26,250 mg of Met (42.9 µg/mL at admission); this patient displayed acute tubular necrosis [7]. In the case a person without renal dysfunction overdosed on Met, he presented with high Met concentrations and consequently with AKI [9]. These results suggest that high Met concentrations possibly cause AKI.

Up to day 19, the patient showed moderate renal failure (eGFR: 59.9–61.2 mL/minute). A previous report indicated that patients with severe and moderate renal failure had similar Met PK parameters [15]. In that report, patients with severe renal failure (*t*_1/2_ = 17.2 hours) who received a single 850 mg Met dose had an estimated *C*_max_ of 3.93 µg/mL [15]. From this *C*_max_, we extrapolated the *C*_max_ of our patient who had taken 500 mg of Met three times a day before admission, when the symptoms started to appear. Using the equation ${C}_{\mathrm{max}}\left(1/\left(1-{e}^{-ke\cdot \tau }\right)\right)\times {C}_{\mathrm{max}}\left(\tau =8\mathrm{h}\right)\times {C}_{\mathrm{max}}\left(\tau =8\mathrm{h}\right)$, we obtained a *C*_ss,max_ of 8.4 µg/mL. This value is lower than our estimated *C*_0_ of 58 µg/mL. This data suggests that other PK parameters elevated the *C*_0_.

Considering the following PK formulas, ${CL}_{\mathrm{tot}}/F={k}_{e}{/V_d}$ and ${C}_{t}=F\cdot {D}_{t}/{V}_{d}$, one PK parameter possibly altering the *C*_0_ is the volume of distribution (*Vd*). That is, a decreased *Vd* can increase the Met plasma concentration levels. Considering the patient’s background, dehydration probably caused by working outside in high temperatures, decreased *Vd* [17]. Moreover, dehydration is a risk factor for AKI [14]. Therefore, dehydration might be an important factor in AKI development with increased Met plasma concentration levels.

Furthermore, in this case, the other possible AKI causes were alcohol consumption and the use of other prescribed medication, such as angiotensin receptor blockers (ARBs) [10, 14]. Alcohol consumption frequently causes dehydration, especially when accompanied with water diuresis [18]. A report on a patient who took 5 g Met and approximately 600 mL Japanese sake developed lactic acidosis; the alcohol consumption rapidly increased the hepatic NADH/NAD^+^ ratio in the patient, which inhibited the gluconeogenesis from lactate [2]. Thus, advised limited alcohol consumption during the use of Met consultation would be recommended, since alcohol can reduce the liver’s capacity to metabolize lactate and induces dehydration [19]. Furthermore, ARBs and SGLT2 inhibitors may cause AKI by decreasing the glomerular filtration pressure, especially in patients that are dehydrated [14, 20]. Nonsteroidal anti-inflammatory drugs (NSAIDs)-induced AKI could be excluded because loxoprofen was topically applied using a hydrogel patch.

The limitations of this study included the scarce Met concentration data and the absence of the quantification of other drugs possibly related to AKI, such as ARBs or azilsartan. Further analyses of the Met concentration in the dialyzed fluid samples can increase our understanding of Met distribution. Moreover, it is difficult to rule out the alternate possibility to increase plasma Met concentration that acute tubular necrosis caused the accumulation of Met in tubular epithelium. For example, multidrug and toxin extrusion proteins (MATEs) are transporters located on the apical side of tubular epithelium [21, 22], involved in the renal secretion of Met [23]. Thus, acute tubular necrosis causes the dysfunction of MATEs, subsequently deteriorating tubular secretion of Met. Further histochemical analysis of Met-related transporters such as MATEs in tubular epithelium would be needed to understand this phenomenon.

To the best of our knowledge, this is the first report of AKI caused by high plasma Met concentration levels with mild lactic acidosis in a patient treated with the recommended Met dose. PK analysis unveiled the importance of dehydration and alcohol consumption to cause high plasma Met by decreasing *Vd*. Other factors, including prescription medication use, might also play a part in the development of AKI. Taken together, patients with several AKI risk factors should be aware that even the daily recommended Met dose could induce AKI.

## Data Availability

The datasets obtained and analyzed during the current study are available from the corresponding author on reasonable request.

## Abbreviations

AKI: Acute kidney injury
ARBs: Angiotensin receptor blockers
BUN: Blood urea nitrogen
CT: Computed tomography
eGFR: Estimated glomerular filtration rate
HD: Hemodialysis
HPLC: High-performance liquid chromatography
MATE: Multidrug and toxin extrusion protein
Met: Metformin
PK: Pharmacokinetic
T2DM: Type II diabetes mellitus
UV: Ultraviolet
